# FRAILTY, FALL RISK, AND FEAR OF FALLING POSITIVELY ASSOCIATED IN COMMUNITY-DWELLING OLDER ADULTS

**DOI:** 10.1093/geroni/igae098.3986

**Published:** 2024-12-31

**Authors:** Abigail Tice, Norma Conner, Wei Zhang, Qun Huo, Yingru Li, Christopher Emrich, Rui Xie, Ladda Thiamwong

**Affiliations:** University of Central Florida, Orlando, Florida, United States; University of Central Florida, Orlando, Florida, United States; University of Central Florida, Orlando, Florida, United States; University of Central Florida, Orlando, Florida, United States; University of Central Florida, Orlando, Florida, United States; University of Central Florida, Orlando, Florida, United States; University of Central Florida, Orlando, Florida, United States; University of Central Florida, Orlando, Florida, United States

## Abstract

More than one in four older adults fall annually, resulting in 3 million emergency department visits. Frailty, a biological syndrome resulting from declining physiological systems, increases vulnerability to adverse outcomes in older adults. Frailty has been identified as a risk factor for fall occurrence in older adults. Many community-dwelling older adults report heightened fear of falling (FOF), and FOF is recognized as a risk factor for fall occurrence. While frailty is significantly, positively correlated with fall risk and FOF, the relationship between all three measures is less understood. The current objective was to examine relationships between frailty, fall risk, and FOF using the Fatigue, Resistance, Ambulation, Illness, and Loss of weight (FRAIL), Stopping Elderly Accidents, Death, and Injuries (STEADI), and short Fall-Efficacy International (short FES-I) questionnaires, respectively, in 178 older adults 65 years or older in Orlando, Florida. Spearman correlation analysis revealed significant associations between FRAIL and STEADI scores (p< 0.0001, r=0.6291), FRAIL and short FES-I scores (p< 0.0001, r=0.5294), and STEADI and short FES-I scores (p< 0.0001, r=0.7230). Linear regression analysis supports the significant associations between FRAIL and STEADI scores (p< 0.0001, r2=0.4407), FRAIL and short FES-I scores (p< 0.0001, r2=0.3293), and STEADI and short FES-I scores (p< 0.0001, r2=0.4636). The significant positive relationships suggest that as frailty increases, fall risk and FOF increase and that as FOF increases, fall risk increases. This work implicates the importance of screening in older adults to prevent the progression of frailty to reduce fall risk and FOF. Addressing FOF is important to reduce fall risk in older adults.

